# Husk to caryopsis adhesion in barley is influenced by pre- and post-anthesis temperatures through changes in a cuticular cementing layer on the caryopsis

**DOI:** 10.1186/s12870-017-1113-4

**Published:** 2017-10-23

**Authors:** M. Brennan, T. Shepherd, S. Mitchell, C. F. E. Topp, S. P. Hoad

**Affiliations:** 1 Scotland’s Rural College, King’s Buildings, West Mains Road, EH9 3JG Edinburgh, Scotland; 2 James Hutton Institute, Invergowrie, DD2 5DA Dundee, Scotland; 3 University of Edinburgh, King’s Buildings, Mayfield Road, EH9 3JH Edinburgh, Scotland

**Keywords:** Caryopsis, Cementing layer, Grain development, Grain skinning, *Hordeum vulgare*, Husk adhesion, Malting barley

## Abstract

**Background:**

At ripeness, the outer husk of “covered” barley grains firmly adheres to the underlying caryopsis. A cuticular cementing layer on the caryopsis is required for husk adhesion, however the quality of adhesion varies significantly among cultivars which produce the cementing layer, resulting in the economically important malting defect, grain skinning. The composition of the cementing layer, and grain organ development have been hypothesised to influence the quality of husk adhesion. Plants of *Hordeum vulgare* ‘Concerto’ were grown at different temperatures pre- and post-anthesis to effect changes in the development of the husk, caryopsis and cuticular cementing layer, to determine how these variables influence the quality of husk-to-caryopsis adhesion.

**Results:**

Warm conditions pre-anthesis decreased the quality of husk adhesion, and consequently increased the incidence of grain skinning. Cool post-anthesis conditions further decreased the quality of husk adhesion. The composition of the cementing layer, rather than its structure, differed with respect to husk adhesion quality. This cementing layer was produced at the late milk stage, occurring between nine and 29 days post-anthesis, conditional on the temperature-dependent growth rate. The compounds octadecanol, tritriacontane, campesterol and β-sitosterol were most abundant in caryopses with high-quality husk adhesion. The differences in adhesion quality were not due to incompatible husk and caryopsis dimensions affecting organ contact.

**Conclusions:**

This study shows that husk-to-caryopsis adhesion is dependent on cementing layer composition, and implies that this composition is regulated by temperature before, and during grain development. Understanding this regulation will be key to improving husk-to-caryopsis adhesion.

**Electronic supplementary material:**

The online version of this article (doi: 10.1186/s12870-017-1113-4) contains supplementary material, which is available to authorized users.

## Background

The mature barley grain comprises the caryopsis (fruit) enclosed in an outer husk which adheres to the caryopsis during grain development, and remains adherent at harvest and during post-harvest handling. If the husk becomes partially or wholly detached, the grain has “skinned”. Grain skinning, also known as “peeling”, is a quality defect for malting barley. During malting, grains undergo a process of controlled germination, during which enzymes “modify” the starchy endosperm by hydrolysing the cell wall and protein matrix, making starch available for fermentation by yeasts during brewing and distilling. Good quality husk adhesion serves to protect the embryo from mechanical damage during harvesting, and processing by maltsters, brewers and distillers. As a viable embryo is essential to the malting process, high levels of grain skinning leads to inefficiencies in malt production. Further, if a grain has skinned but retains a viable embryo, such grains will imbibe water more quickly and may over-modify with respect to intact grains, resulting in malting losses [1–3]. The quality of husk adhesion is influenced by both environmental and genetic factors, and the quality defect grain skinning has increased in severity in recent years with newer varieties being more susceptible to the condition [4]. Ensuring the supply of high quality grain by local growers is essential for the long-term sustainability of malting barley supply chains worldwide, reducing the need to transport grain bulks long distances to make up any quality shortfall. Reducing the risk of grain skinning by increasing the quality of husk adhesion will benefit growers, barley breeders and processors within the malting supply chain.

The barley caryopsis comprises the embryo, the starchy endosperm and the outer aleurone endosperm, surrounded in turn by the nucellar layer, the testa (seed coat), and the pericarp (fruit coat). The outer husk is composed of two glumes, namely the lemma on the dorsal side of the grain, and the palea on the ventral side of the grain [5, 6]. It has been hypothesised that physical damage to malting barley grains, including grain skinning, may be exacerbated by changes in grain length and width, and an incompatibility between grain size and the mechanical strength of the outer husk tissues [7, 8]. Physical contact between the caryopsis and the husk could be expected to differ with changes in grain or glume size. Although there are limited data on differential grain development that relate directly to the husk-caryopsis adhesion process, studies on the effects of temperature differences pre- and post-anthesis on husk and caryopsis development are useful to understand how temperature could be used to manipulate differential growth of these organs and therefore contact between the caryopsis and glumes. For example, higher grain weight attained by increased starch accumulation during growth at low temperatures [9] might increase grain dimensions and result in better contact, and therefore adhesion, between the caryopsis and the husk. Conversely, grains with reduced weights caused by high temperature stress [10, 11] might be expected to have reduced husk-caryopsis contact, resulting in poor quality adhesion. Equally, the size of the husk organs would contribute to the capacity for contact between the husk and caryopsis. Indeed, grain size has been postulated to be determined by the physical limitation of the size of the husk [12, 13], potentially due to effects of pre-anthesis temperature on floret growth [14].

The husk adheres to the underlying caryopsis through a cementing layer, which is thought to be composed of lipids [15–18]. The barley grain contains three internal lipid layers. The thinnest is present in between the nucellar layer and the testa, whereas the thickest is present between the testa and the pericarp. These tissues and the lipid layers develop as a unit. The third lipid layer, the cementing layer, develops after the pericarp cuticle is formed. This cementing layer is 100 to 600 nm thick [15], covering the pericarp cuticle and adhering to the inner husk surfaces later in grain development. This sticky cementing substance is reported to be produced from 10 days after anthesis [15, 17]. However, it has not yet been determined whether this substance is always produced by 10 days after anthesis regardless of the rate of grain development, or whether it is the developmental stage that governs production of the cementing layer. An irregular, reticulate interface exists between the pericarp cell wall and the cuticle during production of the cementing layer, indicating that it is the epidermal cells of the pericarp, rather than the husk, that produces the cementing material [8, 15]. Hull-less, or “naked” barley does not produce a cementing layer, and naturally threshes free of the husk at harvest. Naked barley has a mutation at the *nudum* (*nud*) locus on chromosome 7H, which is homologous to the *WIN1/SHN1* transcription factor gene of *Arabidopsis* thought to regulate a lipid biosynthetic pathway [18]. Although hull-less barley does not produce a cementing layer, it does produce a cuticle on the pericarp surface [15] which is not able to be dyed by Sudan dyes, whereas the caryopses of “covered” barley are dyed by Sudan Black [18]. This suggests that the cementing substance is likely to be similar to, or part of, the solvent-extractable surface lipid layer of the pericarp cuticle.

The location of the cementing layer, either comprising, or lying between, the surface cuticles of the husk and caryopsis, suggest it is likely to be similar in nature and composition to other plant cuticles. Plant surface cuticles are synthesised by epidermal cells and their general structure can be described as having two domains, although it is more commonly being viewed as a lipid-embedded continuation of the cell wall itself [19, 20]. The inner domain is rich in the polymer cutin and is physically associated with the cell wall, often referred to as the “cuticular layer” [21]. The outer domain is rich in wax compounds that are soluble in organic solvents and is termed the “cuticle proper”. The waxy domain has two distinct layers, one with intracuticular waxes embedded in a cutin matrix, and an outer epicuticular layer of waxes that coat the surface and may form crystalline structures. The synthesis of cutin and cuticular wax has been well reviewed [22–26]. Cutin is a polyester of hydroxy and hydroxy epoxy C_16_ and C_18_ fatty acids. Cuticular waxes comprise a complex mixture of compounds including alcohols, alkanes, alkenes, aldehydes, ketones, triterpenes, esters and fatty acids. Biosynthesis of plant cuticular wax is achieved by de novo synthesis of fatty acyl chains from a malonyl-CoA precursor by the fatty acid synthase complex. Fatty acids are then elongated by fatty acid elongase. Further elongation and modification to major wax compounds proceeds via either of two pathways: the decarbonylation pathway or the acyl reductive pathway. The major components of barley leaf surface waxes are primary alcohols, with 1-hexacosanol (C_26_ alcohol) accounting for more than 75% extractable cuticular wax from leaves [27, 28].

Although the composition of plant cuticles differs among organs of the same plant, and even among cultivars [29, 30], the compound classes that comprise the cuticles of different organs are typically the same. The pericarp cuticles of fruit undergo significant compositional changes during fruit development and ripening [31–34]. Like the cuticles of other plant organs, the composition of fruit cuticles is also dependent on environmental factors such as temperature, light, humidity and pathogen attack [35–37]. Cuticle properties such as transpiration rate and permeability, are more highly influenced by their composition rather than by the thickness of the cuticle, or amount of wax. Indeed, mutations in several cuticle-synthesis associated genes alter cuticle permeability, and display an organ fusion phenotype [38–42], which has similarity to the process of husk adhesion, in that adjacent organs adhere to one another through the cuticle [18, 43].

This study used differential temperatures during husk and grain development to separately effect changes in husk and grain size, the structure and composition of the surface cuticles and therefore the cementing layer. It was hypothesised that the quality of husk adhesion, and therefore the severity of grain skinning, would be influenced by one or all of the above, and that by measuring these we would gain insight into critical grain and cuticle developmental stages that influence husk-to-caryopsis adhesion.

## Methods

### Plant growth and sampling

A two-row spring barley (*Hordeum vulgare* cv. Concerto) known to be susceptible to skinning [4] was sown directly in Levingtons No. 2 compost, at a density of seven plants per pot in a total of 36 four-litre pots, and established in a glasshouse until the first leaf came through the coleoptile (GS 10). Developmental growth stages (GS) referred to throughout the study are those of the decimal code described by Tottman and Broad [44]. Thereafter, pots were moved into two Sanyo Fitotron (SGC097 CSX.F) growth cabinets set at day/night temperatures of 18 °C/13 °C, 79% relative humidity, 200 μmol m^−2^ s^−1^ photosynthetically active radiation and grown for 4 weeks under a 10 h photoperiod to reduce tillering [10, 45]. The cabinet conditions were then changed to “cool” or “warm” temperatures set to day/night 13 °C/7 °C or 28 °C/22 °C respectively, with relative humidity 79% and a 16 h photoperiod. The position of the pots within the cabinets was rearranged weekly to reduce positional effects by moving every pot one position to the left each week in the manner of MacNicol et al. [10]. The date of anthesis (GS 65, flowering half-way complete) was determined by visual inspection of the florets. Half of the pots (18) from each cabinet were then switched between the warm and cool cabinets at anthesis, giving a total of nine pots for each of the following four treatments depending on temperatures pre- and post-anthesis, respectively: cool, cool = “CC”; cool, warm = “CW”; warm, warm = “WW” and warm, cool = “WC”. Growth stages were monitored from booting (GS 45) through to harvest-ripeness (GS 92). Thermal time (°C days) for development was calculated using the mean hourly temperature for the cool (16.3 °C) and warm (26.0 °C) cabinets, multiplied by the number of days until that development stage was reached. Three ears from the main shoots were harvested at growth stages 45, 51 and 65 from the two warm and cool pre-anthesis treatments, and at post-anthesis growth stages 75, 77, 85 and 92 from the four treatments listed above. The three ears at each growth stage were harvested from separate pots to ensure spatial replication. The three central florets or grains from one side of each ear were processed for electron microscopy. The five central florets or grains from the opposing side of the ear were used to measure dimensions and fresh weights of the organs (palea, lemma and caryopsis) with a micrometer (accuracy ±0.05 mm) and Mettler Toledo XP6 microbalance (accuracy ±1 μg), and then processed for surface lipid analysis.

A separate experiment was done to examine the caryopsis surface after development of the cementing layer using scanning electron microscopy. Plants of Concerto, and the hull-less variety Nudinka, were grown in pots in a glasshouse as described above. Grains from both cultivars were harvested at GS 77, when in covered barley, the caryopsis is sticky to the touch. These grains were then fixed and processed for scanning electron microscopy as described below.

### Skinning quantification

Grain skinning was assessed using an in-house procedure, where a threshold of 20% or greater husk loss by area was used to distinguished skinned grains from intact grains (less than 20% husk loss) [4]. Remaining ears from the main shoots and tillers were harvested at ripeness and the ear length, floret number, grain number and grain weights per ear were measured. Hand-threshed ears were then further threshed in a Wintersteiger LD 180 laboratory thresher (Wintersteiger AG, Ried, Austria) for 5 s, and grains scored for skinning.

### Transmission and scanning electron microscopy

For transmission electron microscopy of husk material, segments (3 mm long × 1 mm wide × 1 mm thick) were cut from the centre of the lemma, and spanning one vascular bundle from the palea. Segments of the same size were cut from the centre of the dorsal side of the caryopsis, taking care to ensure that the segments excised included all cell layers down to the starchy endosperm, and avoiding the area over the embryo which does not produce a cementing layer. Segments were fixed in 4% (*w*/*v*) paraformaldehyde and 2% (*w*/*v*) glutaraldehyde in 100 mM sodium 1,4-piperazinediethanesulfonic acid (PIPES) buffer (pH 7.2) for 4 h at room temperature, then overnight (18 h) at 4 °C. Fixed tissue was washed three times in 0.1 M sodium cacodylate buffer (pH 7.3) for 10 min each time. Tissue was then post-fixed in 1% osmium tetroxide in sodium cacodylate for 45 min at room temperature, then washed in three 10 min changes of sodium cacodylate buffer. Washed tissue was dehydrated in an aqueous ethanol series (50, 70, 90, and three × 100%) for 15 min each step, and then twice in propylene oxide for 10 min each time. Samples were then embedded in TAAB 812 resin (TAAB laboratories, Berks, England). Sections, 1 μm thick, were cut on a Leica Ultracut ultramicrotome (Leica Microsystems, Milton Keynes, UK), stained with 1% aqueous toluidine blue in 1% borax and viewed on a light microscope to select suitable areas for investigation. Ultrathin sections, 60 nm thick, were cut from selected areas, stained in 1% aqueous uranyl acetate and Reynolds lead citrate then viewed in a Philips CM120 BioTwin transmission electron microscope (Philips Electron Optics, Eindhoven, The Netherlands). Images were taken on a Gatan Orius CCD camera (Gatan, Oxon, UK). The thickness of the inner and outer cuticles (palea and lemma), outer cuticle (caryopsis) and cementing layer (whole grain) was measured using the open-source software Image J [46]. The mean thickness of the cuticular layers for each of the three replicate ears was calculated by taking the mean of five measurements from each of five micrographs per replicate.

For scanning electron microscopy, tissue was cut into 4 mm × 4 mm segments from the dorsal side of the caryopsis and fixed and dehydrated as above. Samples were dried in a Polaron Critical Point Drier (Quorum Technologies Ltd., Lewes, UK), mounted on aluminium stubs, and sputter coated with 20 nm gold palladium in an Emscope SC500A sputtercoater (Emscope, Kent, UK) before examining with a Hitachi S-4700 scanning electron microscope (Hitachi, Japan).

### Surface lipid analysis

Surface lipid extracts were prepared from the husks (pooled paleas and lemmas) and caryopses of five central grains from replicate ears as described above. Organs were dipped in dichloromethane (puriss. p.a. grade for GC ≥99.9%, Sigma-Aldrich, UK) for 20 s at room temperature and the extract evaporated to dryness under N_2_ (British Oxygen Company, 99.995%). Extracts were re-solubilised in isohexane (HPLC Plus grade for GC ≥98.5%, Sigma-Aldrich, UK) containing 50 ppm BHT (2,6-di-tert-butyl-4-methylphenol) (Sigma-Aldrich, UK) and evaporated to dryness as above. Methyl nonadecanoate (0.5 μg) (Sigma-Aldrich, UK) was added to each sample as an internal standard. Compounds in the extracts with free hydroxyl and carboxyl groups were derivatised to TMSi ethers and esters by addition of 25 μl *N*-*O*-bis-trimethylsilyltrifluoroacetamide (BSTFA, ThermoScientific, UK) and 25 μl anhydrous pyridine (Sigma-Adrich, UK) at 50 °C for 90 min with agitation every 30 min. Wax constituents were analysed by gas chromatography-mass spectrometry (GC-MS) using a Trace DSQ™ II Series Quadrupole system (Thermo Electron Corporation, Hemel Hempstead, UK), fitted with a CTC CombiPAL autosampler (CTC Analytics, Switzerland). Samples (1 μl) were injected into a programmable temperature vaporising (PTV) injector operating in splitless mode and fitted with a Merlin Microseal™ High Pressure Septum and a Siltek™ deactivated metal PTV liner (120 mm × 2 mm internal diameter × 2.75 mm external diameter, Thermo Scientific, UK). The PTV conditions were injection temperature 132 °C for 1 min, transfer rate 14.5 °C s^−1^, transfer temperature 320 °C for 1 min, clean rate 14.5 °C s^−1^ and clean temperature 400 °C for 2 min. Chromatography was effected on a DB5-MS™ column (15 m × 0.25 mm × 0.25 μm; Agilent Technologies, UK) using helium at 1.5 ml min^−1^ (constant flow). The GC temperatures were 100 °C for 2.1 min, 25 °C min^−1^ to 320 °C, then isothermal for 3.5 min. The GC-MS interface temperature was 325 °C. Mass spectrum acquisition conditions were electron impact (EI) ionisation at 70 eV, solvent delay 1.3 min, source temperature 230 °C, mass range 35 to 900 a.m.u. at 6 scans s^−1^. Acquisition rates were set to give approximately 10 data points across each chromatographic peak. Data were acquired and analysed using Xcalibur™ 2.0.7 (Thermo Electron Corporation, Hemel Hempstead, UK). Specific ions characteristic of each compound in the husk and caryopsis samples, including the internal standard (IS), were selected following examination of total ion chromatograms (TIC) for several raw data files of both types of sample. Ion selection was on the basis that they should have as high a relative abundance as possible and should be unique to the compound and/or be well resolved from other ions with the same *m*/*z* [47]. These ions were used for compound detection and quantification in a processing method created in Xcalibur™. For each compound a time window was defined, centred on the chromatographic peak apex and a summed selected ion chromatogram (SIC) for all of the chosen ions was generated within the time window. Response ratios for each analyte were calculated relative to the IS using the calculated SIC areas for both components. Processed data were checked for correct peak assignment and adjusted where necessary. Compounds were identified by comparison of their mass spectra and retention times with reference standards, MS libraries (Palisade 600 k, Palisade Corporation, USA; NIST05, National Institute of Standards, USA), by comparing with retention and MS data for known compounds and by reference to published data. A total of 121 compounds were identified, the masses used for compound identification and quantification are given in Additional file 1: Table S1. The “abundance” of each compound was calculated from the summed relative response of the selected ions for that compound, divided by the number of organs extracted for that sample; the abundance of each compound was then used for statistical analysis as below, abundance data are given in Additional file 1: Table S2.

For each class of compound present in the samples, specific ion groups were selected as follows:

Fatty acids: Two characteristic ion groups were used for identification and quantification of fatty acids as their TMS ester derivatives. The intense ion group *m/z* 117, 129, 132 and 145 are common to all fatty acids and for most acids were used for quantification. The prominant molecular ion [M]^+^ and [M-15]^+^ ions were used to confirm identification of individual acids. However, 14-methylhexadecanoic acid co-eluted with 8-heptadecenoic acid, and both contribute to *m/z* 117, 129, 132 and 145. The ratio of the *m/z* 117, 129, 132, 145 ion group to [M]^+^ plus [M-15]^+^ for the earlier eluting 10-methylhexadecanoic acid was calculated. Assuming the same ratio for 14-methylhexadecanoic acid, the abundance of [M]^+^ and [M-15]^+^ ions for this compound were used to estimate its abundance of *m/z* 117, 129, 132 and 145, and hence the abundance of *m/z* 117, 129, 132, 145 due to 8-heptadecenoic acid. Octacosanoic acid co-elutes with the TMS derivative of campesterol, both of which share *m/z* 129. Consequently, only ions *m/z* 117 and 132 were used for measurement of the abundance of octacosanoic acid, and the measured abundance was multiplied by a correction factor of 1.76 to account for the absence of *m/z* 129 and 145. The correction factor was deduced from data for campesterol-free octacosanoic acid.

Fatty alcohols: The characteristic homologue-specific [M-15]^+^ ions were used for measurement of the abundance of the TMS ether derivatives of long chain alcohols.

Wax esters: The long chain alky esters were identified using two characteristic ions, the molecular ion [M]^+^ from which the overall carbon number could be deduced, and the prominent McLafferty rearrangement ion [RCO_2_H_2_]^+^ arising from the acid portion of the intact ester from which the carbon numbers of the individual acid:alcohol combination could be deduced [48]. Abundance measurements were based on the McLafferty ion.

Alkanes and alkenes: The identity of the most prominent alkane and alkene homologues were determined from their molecular ions [M]^+^ from which the identities of the minor homologs could be deduced by interpolation. Abundance measurements were based on the characteristic series of fragment ions of mass [C_n_H_2n+1_]^+^ for alkanes and [C_n_H_2n-1_]^+^ for alkenes which were common to all homologues.

Ketones, ß-diketones, enols and hydroxy-ß-diketones: Mass spectral fragmentation patterns for these compounds are shown in Additional file 2, in which diagnostic Ions seen in the mass spectra are marked with an asterisk and those used for quantification are underlined. The mass spectra of nonacosan-14-one and hentriacontan-14, 16-dione are dominated by ions arising from fragmentation α or ß to the carbonyl group, the latter also involving hydrogen transfer, typical of ketones and ß-diketones [49, 50]. In the MS of ß-diketones a prominant ion of *m/z* 100 forms from sequential fragmentations on opposite sides of the molecule ß to the diketo group. Other diagnostic fragmentations include loss of 18 from the molecular ion or other fragments. In Additional file 2 diagnostic ions seen in the mass spectra are marked with an asterisk and those used for quantification are underlined. Mass spectral fragmentation schemes for the different enol and (enol)_2_ tautomers of hentriacontan-14, 16-dione are dominated by ions arising from fragmentation α or ß to the carbonyl or OTMS groups, with fragments incorporating OTMS being favoured [51]. The mass spectra of the four enols appear to be very similar and it was not possible to distinguish between them on the basis of their mass spectra since the same ion groups were used for identification and quantification. The same was the case for the three (enol)_2_ tautomers. Rather than attempting to separate the selected ion chromatogram trace for the chosen ions into separate portions for each tautomer, the whole trace was integrated to provide an abundance measurement for all tautomers in combination. Finally, the abundance measurements for the enol and (enol)_2_ components were combined with that for hentriacontan-14, 16-dione to provide an overall abundance measurement for the ß-diketone. Fragmentation α to the OTMS group gives rise to the major diagnostic ions in the mass spectra of 8- and 9-hydroxyhentriacontan-14, 16-diones, although some ions arising from fragmentation to the carbonyl groups are also evident.

5-Alkyl resorcinols: Members of the two homologous series of 5-alky resorcinols are distinguished by the intense fragment ions at *m/z* 268 for unsubstituted homologues and *m/z* 282 for methyl substituted homologues in the mass spectra of the diTMS derivatives, arising from fragmentation between C1 and C2 of the 5-alkyl chain [52, 53]. Individual homologues are identified from their relatively intense molecular ions. The exact position of the methyl group in the methyl substituted compounds cannot be distinguished from the mass spectra, but must be either within the aromatic ring or at C1 of the alkyl side chain.

Terpenes: Ions characteristic of each of the terpenes including free squalene and cholesta-3, 5-diene and sterols and γ-tocopherol as TMS ethers were used for compound identification and characterisation. For most sterols, *m/z* 129 was used as one of the ions, but was excluded for campesterol due to co-elution with octacosanoic acid.

### Statistical analysis

The data were analysed using the open-source software R [54]. The fit of models described below were checked by plotting residuals against fitted values, and also by plotting fitted values against observed values. The effect of treatment on skinning severity was analysed by fitting a generalized linear model [55] to the binomial counts of skinned and total grains for each ear, using “treatment” as the predictor variable. Calculation of 95% profile likelihood confidence intervals was used to determine significant differences among treatments. The effect of the four treatments on ear measurements at harvest ripeness was determined by analysis of variance (ANOVA) (α = 0.05) followed by post-hoc Tukey’s HSD tests (α = 0.05) where a significant effect was found. During plant development, the effect of treatment and growth stage on organ weights and dimensions were analysed separately for three phases of development: husk development (pre-anthesis), grain development and husk adhesion. For each phase a generalized linear mixed effects model was built with the measurement as the response variable, treatment, growth stage and their interaction as fixed effects, with the random effect being ear nested within pot. The final minimally-adequate models were selected by dropping non-significant variables (α = 0.05) on comparison of hierarchical models using ANOVA, and significant differences among samples determined by least squares means comparisons [56]. The effect of treatment and growth stage on the composition of surface lipid extracts was tested for each organ type (husk and caryopsis) separately using a linear mixed effects model. Compound abundance was the response variable, with treatment, growth stage and their interaction as fixed effects, and pot number as the random effect. Hierarchical models were compared by ANOVA and non-significant terms (α = 0.05) were sequentially dropped from each model to find the minimally adequate model for each compound. Significant differences in compound abundance among samples were determined from least-squares means contrasts (α = 0.05).

## Results

### Plant growth

**Fig. 1:**
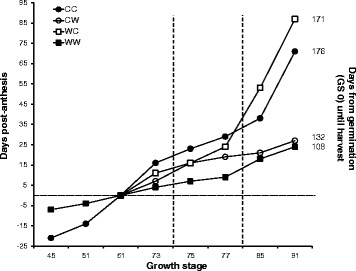
Developmental stage of *Hordeum vulgare* cv. Concerto, in relation to anthesis date (dotted line), grown under four different treatments depending on pre- and post-anthesis temperatures. Number of days from sowing until harvest is displayed on the right hand side. The critical developmental stages for husk adhesion fall within the dashed lines

Development of main shoots and tillers were recorded every few days from booting until ripening and are shown in relation to date of anthesis, which is indicated by a horizontal dotted line in Fig. 1. Ears from plants grown under warm conditions pre-anthesis took an average of 84 days (2184 °C days) from sowing until anthesis, whereas ears from plants grown under cold conditions took an average of 105 (1715 °C days) days to reach anthesis. Development rates also differed among the four post-anthesis treatments, with plants grown in cool post-anthesis conditions taking longer to reach ripening than those grown in warm post-anthesis conditions. The CC plants took 71 days from anthesis to ripening (1160 °C days), the WC plants 87 days (1421 °C days), the WW plants 24 days (624 °C days) and the CW plants 27 days (702 °C days). Across all treatments, the caryopsis became sticky to the touch at GS 77, when the caryopsis is nearing maximum volume. The CC plants took 29 days from anthesis to GS 77 (474 °C days), the WC plants 24 days (392 °C days), the WW plants 19 days (494 °C days) and the CW plants only 9 days (234 °C days). These data suggest that the growing conditions pre-anthesis have an influence on the subsequent rate of development, and length of grain-filling period of the caryopsis. The metabolic processes responsible for production of the sticky cementing material are likely to come into play shortly before the caryopsis becomes noticeably sticky, therefore the period between GS 75 and GS 77 is marked by vertical dashed lines in Fig. 1 to indicate the developmental period, and range in days after anthesis, that the critical period for husk adhesion is likely to span.

### Effect of temperature on grain skinning

**Fig. 2:**
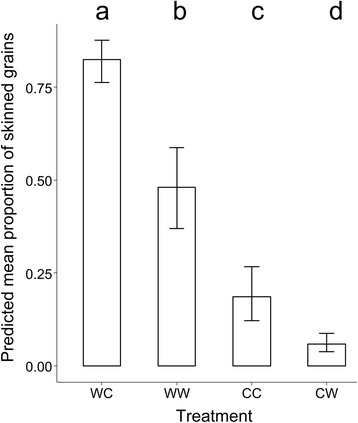
Mean proportion skinned grains per ear for each of the four treatment types. A high proportion of skinned grains indicates poor quality adhesion of the husk to the caryopsis. Significant differences among treatments were determined by comparison of 95% profile likelihood confidence intervals (error bars), and are denoted by different lowercase letters

Both pre-anthesis and post-anthesis temperatures had a significant impact on skinning, with all treatments having significantly different proportions of skinned grains per ear (*P* < 0.001). Differences in skinning among treatments as determined by comparing confidence intervals from the generalised model are shown in Fig. 2. Ears of plants grown in warm pre-anthesis conditions had a higher proportion of skinned grains than those grown in cool conditions pre-anthesis, with the WC plants having the highest proportion of skinned grains (> 0.75).

### Grain development

Measured weights and dimensions of the grain components in the five central florets, specifically the caryopsis and the two glumes of the husk (palea and lemma), are summarised in Table 1. For comparisons of significant differences among measurements, the data were separated into three developmental phases: husk development, grain development, and post-adhesion. Linear mixed effects models were compared to determine the significance of treatment, growth stage and their interaction on each measurement within these three phases. The final, minimally adequate models were determined by comparing hierarchical models using ANOVA, dropping one variable each time (Additional file 1: Table S3). Predicted means and standard error of the difference between means from the minimally adequate models are given in Additional file 1: Table S4.

**Table 1:** Dimensions^a^ and fresh weights^b^ of organs at selected growth stages across four treatments

Phase			Measurement								
Husk development	Treatment	GS	Palea length	Palea width	Palea weight	Lemma length	Lemma width	Lemma weight	Caryopsis length	Caryopsis width	Caryopsis weight^c^
	W	45	8.93 ± 0.39	4.28 ± 0.25	2.58 ± 0.24	10.13 ± 0.33	5.45 ± 0.33	5.55 ± 0.29	–	–	–
	W	51	9.56 ± 0.17	4.73 ± 0.15	3.12 ± 0.02	10.57 ± 0.18	5.69 ± 0.41	6.20 ± 0.65	–	–	–
	W	65	9.44 ± 0.30	4.81 ± 0.37	2.48 ± 0.11	10.45 ± 0.24	5.82 ± 0.19	4.96 ± 0.30	–	–	–
	C	45	8.68 ± 0.47	4.46 ± 0.15	2.46 ± 0.43	9.48 ± 0.42	6.30 ± 0.04	5.29 ± 0.65	–	–	–
	C	51	9.42 ± 0.11	4.90 ± 0.04	2.67 ± 0.22	10.14 ± 0.11	6.64 ± 0.13	5.43 ± 0.10	–	–	–
	C	65	9.83 ± 0.20	5.07 ± 0.08	2.62 ± 0.08	10.74 ± 0.23	6.69 ± 0.15	5.84 ± 0.28	–	–	–
Grain development											
	WC	75	9.86 ± 0.16	5.33 ± 0.13	2.81 ± 0.24	10.60 ± 0.14	6.55 ± 0.14	5.88 ± 0.41	8.45 ± 0.17	3.00 ± 0.10	32.56 ± 1.32
	WC	77	10.13 ± 0.15	5.03 ± 0.20	2.98 ± 0.22	10.74 ± 0.22	6.52 ± 0.09	6.03 ± 0.13	9.07 ± 0.13	3.56 ± 0.12	48.87 ± 5.07
	WW	75	10.49 ± 0.34	5.08 ± 0.12	2.97 ± 0.27	10.71 ± 0.34	6.34 ± 0.23	5.70 ± 0.54	9.65 ± 0.38	3.37 ± 0.39	35.21 ± 2.73
	WW	77	10.01 ± 0.20	5.21 ± 0.16	2.98 ± 0.13	10.26 ± 0.76	6.38 ± 0.37	6.04 ± 0.36	8.53 ± 0.39	3.69 ± 0.05	50.23 ± 1.28
	CC	75	10.12 ± 0.07	4.88 ± 0.07	2.69 ± 0.02	10.78 ± 0.31	6.72 ± 0.04	5.91 ± 0.07	9.94 ± 0.12	3.34 ± 0.21	45.69 ± 5.59
	CC	77	9.87 ± 0.31	4.56 ± 0.06	2.68 ± 0.21	10.32 ± 0.31	6.74 ± 0.13	5.87 ± 0.56	9.59 ± 0.28	4.13 ± 0.09	64.96 ± 2.14
	CW	75	9.52 ± 0.18	4.60 ± 0.05	2.33 ± 0.08	10.04 ± 0.10	6.40 ± 0.10	4.58 ± 0.23	8.88 ± 0.32	3.10 ± 0.13	35.54 ± 3.87
	CW	77	9.62 ± 0.19	4.24 ± 0.20	2.44 ± 0.14	9.83 ± 0.18	6.44 ± 0.29	4.58 ± 0.40	8.63 ± 0.01	3.36 ± 0.07	39.34 ± 1.26
Post-adhesion											
	WC	85	–	–	–	–	–	–	9.83 ± 0.22	4.24 ± 0.04	76.42 ± 4.75
	WC	92	–	–	–	–	–	–	7.41 ± 0.21	3.40 ± 0.08	46.24 ± 3.63
	WW	85	–	–	–	–	–	–	9.80 ± 0.17	4.18 ± 0.00	75.29 ± 0.92
	WW	92	–	–	–	–	–	–	8.05 ± 0.15	3.43 ± 0.09	41.07 ± 1.52
	CC	85	–	–	–	–	–	–	9.49 ± 0.09	4.62 ± 0.13	94.63 ± 6.02
	CC	92	–	–	–	–	–	–	8.43 ± 0.18	4.03 ± 0.02	68.73 ± 2.84
	CW	85	–	–	–	–	–	–	9.48 ± 0.08	4.30 ± 0.02	77.96 ± 3.11
	CW	92	–	–	–	–	–	–	8.08 ± 0.38	3.46 ± 0.05	47.39 ± 3.54

Values are expressed as mean ± standard error of the mean

^a^Measurement in mm

^b^Measurement in mg

^c^Note that at GS 85 and GS 92, caryopsis measurements include the palea and lemma and are therefore more accurately “grain” measurements

“-” Indicates that measurements could not be made due to the growth stage

During husk development (ie pre-anthesis, GS 45, 51 and 65), only two temperature treatments could be compared, warm (W) and cool (C). Growth stage had a significant effect on palea length (*P* < 0.001), width (*P* = 0.021), and weight (*P* = 0.013). At GS 41, paleas were shorter and less wide than at GS 51 or 65. Treatment had no effect on palea weights or dimensions. For lemma length, GS was the only significant variable (*P* = 0.017), but post-hoc tests indicated no significant differences among samples. Lemma width on the other hand was significantly affected by treatment only (*P* < 0.001), with C plants having wider lemmas than W plants. Although the interaction between treatment and GS was significant for lemma weight (*P* = 0.049), post-hoc tests showed no significant differences among samples.

During grain development (GS 75 and 77), treatment significantly affected palea length (*P* = 0.011) width (*P* < 0.001), and weight (*P* = 0.003), with GS also being a signficant factor of palea width (*P* = 0.022). Palea length was highest in the WW plants compared with the other three treatments. Within treatments, there was no difference between palea widths between GS 75 and 77, but both growth stages in WC plants had wider paleas than those in CW plants. Palea weight was greater in the CW plants than in WC or WW. Treatment did have a significant effect on lemma weights (*P* < 0.001), with CW lemmas weighing significantly less than those grown under WC, WW or CC conditions. The interaction between GS and treatment was significant for both caryopsis length (*P* < 0.001) and weight (*P* = 0.047), with caryopsis width being affected by treatment (*P* < 0.001) and GS (*P* = 0.014). WW caryopses were significantly shorter, wider and heavier at GS 77 than GS 75. Caryopsis width also increased significantly between GS 75 and 77 for the WC, CC and CW treatments. Only CC and WW plants had a significant increase in caryopsis weight between GS 75 and 77.

During the post-adhesion phase, the husk could not be removed from the caryopsis without causing damage to the husk, or the underlying tissues of the caryopsis; “caryopsis” measurements therefore include the husk organs, and are more accurately “grain” measurements. Growth stage had a significant effect on grain width (*P* < 0.001) and weight (*P* < 0.001). Treatment also had a significant effect on grain width (*P* < 0.001) and weight (*P* < 0.001), with the interaction between treatment and growth stage significantly affecting grain length (*P* = 0.004). For all treatments, grain length and width decreased between GS 85 and ripeness (GS 92), with CC plants having wider grains at harvest ripeness than the other treatments. Grain weight also significantly decreased between GS 85 and 92 for all four treatments, with CC plants having significantly heavier grains than the other three treatments. Treatments with the highest or lowest grain weight, length or width at harvest did not correspond to the treatments that induced the highest or lowest skinning.

### Ear measurements at harvest

Measurements of harvest ripe ears are summarised in Table 2. Temperature had a significant effect (*P* < 0.001) on all measured ear traits at harvest. The conditions pre-anthesis determined ear length and grain number, with plants grown in warm conditions pre-anthesis having significantly shorter ears, with a lower floret number and high infertility resulting in a significantly lower number of grains compared with plants grown in cool conditions pre-anthesis. Within each pre-anthesis temperature, there were no significant differences in ear length, floret number or grain number between the different post-anthesis treatments. Grain weight on the other hand, was significantly affected by the post-anthesis temperatures. Grain weight was highest in ears from CC plants, with the other three treatments not having significantly different grain weights from each other.

**Table 2:** Differences^a^ in harvest-ripe ears among treatments

Treatment	N	Ear length (mm)	Floret number^b^	Grain number^b^	Grain weight (mg)	% Infertility
WC	37	6.1 ± 0.2 a	19.1 ± 0.6 a	4.7 ± 0.6 a	43.5 ± 2.0 a	75.4
WW	92	5.8 ± 0.1 a	17.3 ± 0.4 a	7.3 ± 0.5 a	43.6 ± 1.9 a	57.8
CC	37	7.5 ± 0.2 b	28.8 ± 0.5 b	15.5 ± 1.2 b	66.9 ± 1.0 b	46.2
CW	96	7.6 ± 0.1 b	29.2 ± 0.4 b	22.7 ± 0.9 b	37.4 ± 1.2 a	22.3

^a^Values are expressed as mean values for each ear ± standard error of the mean. Significant differences among treatments (post-hoc Tukey’s HSD, α = 0.05) are designated by lowercase letters

^b^These are mean values and therefore not integers

### Structure of the husk and pericarp cuticles, and the cementing layer

**Fig. 3:**
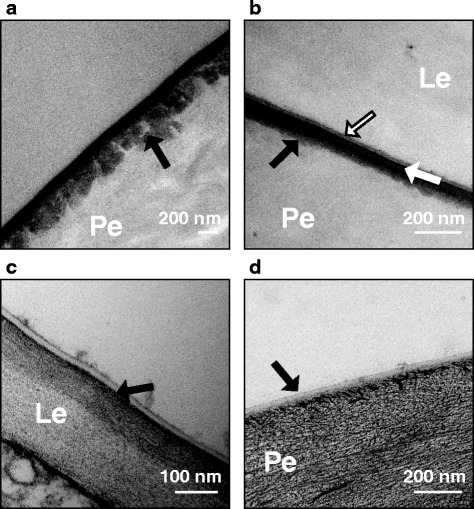
Transmission electron micrographs of husk and caryopsis surface cuticles. **a** Caryopsis at GS 75 from WC treatment. There is an electron-dense cuticle proper and underlying flocculate cuticular layer (black arrow). **b** Well-adhered cementing layer at GS 85 from CC treatment. The cementing layer comprises the caryopsis cuticle (black arrow), and the inner cuticle of the husk (black and white arrow). Alternating electron-dense and electron-lucent lamellae are present between the cuticles (white arrow). **c** Inner surface cuticle of a lemma at GS 45 grown in warm conditions. The interface between the electron-lucent cuticle and cell-wall of the lemma is smooth (black arrow). **d** Nudinka caryopsis at GS 77. A thin, electron-lucent surface cuticle is present (black arrow). Le = lemma cell wall, Pe = pericarp cell wall

The structure of the cuticular layers and cementing layer was similar across all treatments, but differed among growth stages. A light micrograph of a barley grain section post-adhesion is provided in Additional file 3, showing a low-powered orientation of the husk and caryopsis tissue organisation. At GS 75, there was typically an irregular interface between the electron-dense cuticle proper and the pericarp cell wall, signifying ongoing production of cuticular material (Fig. 3a, arrow). At GS 77, the interface between the cuticle and the cell wall was often smoother in appearance, although the cuticle proper was still typically electron-dense. By GS 85, after husk adhesion is complete, production of further cuticular material had ceased, and the cuticular layer comprised the pericarp cuticle, the husk cuticle and a layer of material in between that was either amorphous and electron dense, or a lamellated structure with alternating layers of electron dense and electron translucent material (Fig. 3b). The inner and outer surfaces of the husk cuticles do not undergo increased production of cuticular material during critical periods of husk adhesion, and do not have a lamellated structure at any developmental stage when the husk can still be removed from the pericarp. An example of the inner lemma surface cuticle at GS 45 is shown in (Fig. 3c). The pericarp surface cuticle of the hull-less variety “Nudinka” at GS 77 was examined for comparative purposes, and was found to have a thin cuticular layer of ~30 nm which is shown in Fig. 3d. The interface between the cuticle and the pericarp cell wall of Nudinka is smooth, with no indication of continuing production of cuticular material.

**Fig. 4:**
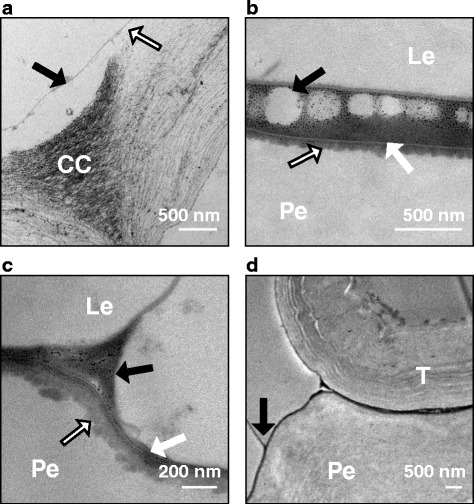
Transmission electron micrographs of husk and caryopsis cuticles at regions of poor adhesion. **a** Inner surface cuticle of a palea at GS 51 grown in cool conditions. The cuticular layer (solid black arrow) often began to detach from the underlying cell wall (detachment shown by black and white arrow) at the corners between adjacent epidermal cells. **b** Region of separation between the husk (lemma) and pericarp at GS 85. The cementing layer does not fill the entire space between the pericarp and the husk, evidenced by gaps in the cementing material (black arrow). The interface between the pericarp cell wall and the pericarp cuticle is not smooth, but does not show globular deposits of cutin (black and white arrow). A thin, electron-lucent outer cuticle separates the pericarp cuticle from the electron-dense cementing material (white arrow). **c** Caryopsis at GS 85 from CC treatment. The surface cuticles of the husk and caryopsis are separated (black arrow). The underlying cuticular layer of the pericarp has a slightly flocculate appearance (black and white arrow) with electron-dense lamellae in the cuticle proper (white arrow). At the point of separation, these lamellae are not all in parallell, but occur at several orientations. Lamellae do not occur on the inner cuticle of the lemma. **d** Tubelike cell at a region of separation between the husk and caryopsis (black arrow) at GS 85 in a CC grain. CC = cell corner, Le = lemma cell wall, Pe = pericarp cell wall, T = tubelike cell

The surface cuticles often became separated from the underlying cell wall in both the husk and pericarp samples, typically at the corners where adjacent epidermal cells join (Fig. 4a, arrow), although this separation sometimes extended across the whole sample. From GS 77 onwards, the pericarp and husk cuticles began to adhere together, and were sometimes seen to be detached from both husk and pericarp cell walls, but remaining firmly attached to each other. From GS 85, large spaces between the cuticles often had an electron dense layer between them which did not always extend to fill the space between the husk and pericarp when the separation distance was particularly large (Fig. 4b). This electron dense material later became lamellated with either parallel striations, or lamellations with random orientations at regions of husk-caryopsis separation (Fig. 4c). These lamellations measured between four to 10 nm thick. Separation of the cuticular layers was common in regions adjacent to “tubelike” cells, which are most likely trichomes, and potentially cause some mechanical stress between the husk and caryopsis (Fig. 4d). Lamellae were not observed in cuticles of either the inner or outer surfaces of the palea or lemma.

The frequent separation of the cementing layer from the pericarp and husk cell walls during the adhesion phase made it difficult to measure (shown in Additional file 3), with only a small number of samples retaining sufficiently intact cementing layers to calculate the standard error of the mean (Table 3). Pre-husk adhesion, the inner and outer cuticles of the husk organs were thinner than the pericarp surface cuticle. From GS 85, after husk adhesion is complete, the inner husk cuticle was measured together with the pericarp cuticle as part of the entire cementing layer, with the entire layer measuring approximately 100 nm in thickness. In regions of evident husk-caryopsis separation however, the cementing layer could be up to several hundred nm in thickness.

**Table 3:** Measurement of surface cuticle thickness of grain components

Treatment	GS^a^	Palea inner	Palea outer	Lemma inner	Lemma outer	Caryopsis/pericarp
W	51	42.90 ± 13.01	25.88 ± 7.68	24.87 ± 10.42	52.97 ± 6.03	–
C	51	28.54 ± 14.58	51.64	27.80	62.58 ± 5.74	–
W	65	25.38 ± 4.37	31.87 ± 4.39	36.67 ± 7.12	35.63 ± 4.84	–
C	65	37.83 ± 6.85	62.84 ± 7.66	26.30 ± 1.22	67.43 ± 4.40	–
WC	75	34.15 ± 5.00	35.27 ± 1.14	48.16	22.87 ± 9.32	76.29 ± 20.67
WW	75	30.89 ± 2.69	51.42 ± 22.18	34.41 ± 6.77	79.68 ± 11.08	83.27 ± 6.56
CC	75	39.85 ± 3.04	52.40	32.78 ± 8.23	m.v.	90.12
CW	75	17.30 ± 5.36	m.v.	43.87	m.v.	53.44
WC	77	m.v.	25.34 ± 1.26	36.55 ± 13.25	86.83 ± 44.67	93.70 ± 45.86
WW	77	32.59 ± 3.01	27.22 ± 5.02	30.93 ± 1.96	83.80 ± 33.69	82.62 ± 12.48
CC	77	38.45 ± 6.43	27.00	31.07 ± 1.34	58.80	91.40
CW	77	59.04	24.96	30.99 ± 19.85	m.v.	50.33
WC	85	–	–	–	–	118.98
WW	85	–	–	–	–	84.06
CC	85	–	–	–	–	113.12 ± 20.00
CW	85	–	–	–	–	131.22 ± 16.20

Where three samples could be measured, values are expressed as mean ± standard error of the mean; where fewer than three samples could be measured, only the mean is given

“-” Indicates that measurements were not made due to the growth stage

“m.v.” Indicates missing data due to cuticle separation from cell walls

^a^Note that at GS 85, caryopsis measurements comprise the husk and pericarp cuticles, whereas only the pericarp is measured on the caryopsis surface at earlier growth stages

### Pericarp surface morphology

**Fig. 5:**
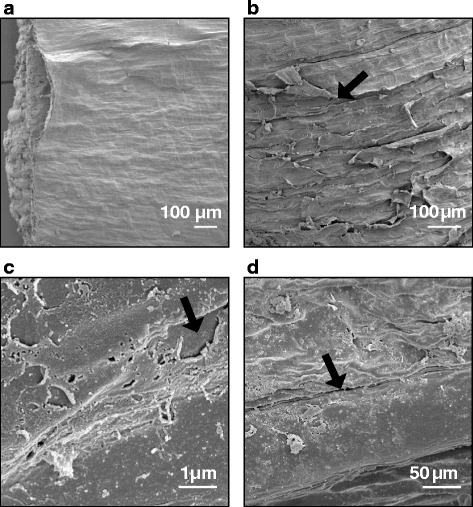
Scanning electron micrographs of caryopsis surfaces at GS 77. **a** The pericarp surface of Nudinka at GS 77 is smooth and un-damaged. **b** The pericarp surface of Concerto has been damaged by removal of the husk, evidenced by broken cells (black arrow). **c** At higher magnification the surface of the cementing material can be seen. It is a layer of globular material that has been damaged by removal of the husk. Where the cementing material has been pulled away, the underlying surface is smooth (black arrow). **d** The junction between a tubelike cell and the pericarp surface is shown (black arrow), demonstrating that the tubelike cell is not covered by the globular cementing material

As the surface cuticles were often observed to have pulled away from the cell walls, grains of Concerto at GS 77 were examined by scanning electron microscopy to determine whether this separation was likely to be happening during physical removal of the husk from the caryopsis, or during sample processing. The hull-less variety “Nudinka” was examined at GS 77, the growth stage at which the pericarp surface becomes sticky in covered varieties, to compare pericarp surfaces between grains that produce a cementing layer with a grain that does not. At GS 77, the surface of the Nudinka pericarp is smooth (Fig. 5a, Additional file 3), whereas the surface of the Concerto pericarp is damaged from the action of pulling the husk away from the already adhesive pericarp surface (Fig. 5b). The surface of Concerto at GS 77 is covered by a further layer of globular material Fig. 5 c), most likely the cementing material, which does not smoothly coat the entire pericarp surface, possibly due to disruption by removal of the husk at this stage. The tubelike cells on the pericarp surface are not fully covered over by the cementing material (Fig. 5d) in agreement with transmission electron microscopy results which show the cementing layer does not entirely fill the spaces between the pericarp and husk around these cells (Fig. 4d). The extent of surface damage in grains of Concerto at GS 77, combined with the absence of damage to Nudinka at GS 77, indicate that separation of the cementing layer from the cell walls is likely to be due to the strength of the adhesion through the cementing layer being higher than that between the cuticle and the cell wall, rather than an artefact of sample processing.

### Surface lipid composition

In the surface lipid extracts from the husk and caryopses, a total of 121 different compounds were identified, belonging to the following structural classes: fatty acids; fatty alcohols; wax esters; alkanes and alkenes; ketones, diketones, hydroxydiketones and related compounds; 5-alkylresorcinols; and triterpenes. For each structural class except the esters, abundances of compounds in each sample are shown by carbon-number distribution in Additional file 4. A total of 20 fatty acids were present, mostly saturated straight chain homologues (even carbon numbers of C_14_ to C_32_, and odd carbon numbers of C_15_, C_17_ and C_25_). Two branched chain C_17_ acids were identified as 14- and probably 10-methylhexadecanoic acids on the basis of their equivalent chain lengths of 16.72 and 16.40 [48, 57]. Of the 14 long-chain alcohols detected, the most abundant were even carbon homologues (C_16_ to C_28_), with odd carbon compounds (C_21_ to C_31_) as minor constituents. A total of 37 long chain esters in the range C_32_ to C_48_ were detected as minor components which consisted of fatty acids (C_14_ to C_24_) esterified to alcohols (C_16_ to C_30_ and C_21_ to C_25_). Among the total of 22 aliphatic hydrocarbons detected, odd carbon alkanes (C_21_ to C_37_) were most abundant with even carbon homologues (C_22_ to C_38_) and odd (C_27_ to C_31_) and even (C_30_) carbon alkenes as minor constituents. Nonacosan-14-one, the β-diketone hentriacontan-14, 16-dione, and both 8- and 9-hydroxy-hentriacontan-14, 16-dione were detected. In addition, several components were detected which appear to be consistent with 4 individual enol tautomers and 3 individual (enol)_2_ tautomers of hentriacontan-14, 16-dione. The interrelationship between these is shown in Additional file 5. Such compounds are not usually reported as being constituents of plant waxes along with ß-diketones and it is unclear whether these components were genuine constituents within the wax, or were artefacts of wax extraction or derivatisation. Keto-enol tautomerism can be both acid and base catalysed, and it is possible that the presence of pyridine in the derivatisation medium facilitated the reaction. However, we have evidence to suggest that the enol and (enol)_2_ forms were also present in the absence of pyridine (data not shown). Since these tautomers are all considered to be derived from hentriacontan-14, 16-dione, their abundances were combined and included within that of the diketone. Two homologous series of 5-alkyl resorcinols were detected. In the first the alkyl substituents consist of odd carbon straight chains (C_15_ to C_27_), whereas in the second the molecule carries an additional methyl substituent, either within the benzene ring or on C1 of the predominantly odd carbon 5-alkyl side chain (C_15_ to C_27_, and C_20_). Six terpenes were detected including the sterol precursor squalene, cholesterol, campesterol and ß-sitosterol, a component tentatively identified as cholesta-3, 5-diene and γ-tocopherol. In addition, 6 compounds of unknown identity were present in the samples.

No compounds were identified that were unique to either the husk or caryopsis samples, treatments or growth stages, although alkylresorcinols and ketones were typically most abundant in the husk extracts. There were significant differences in the abundance of several compounds among treatments and growth stages as described below. Table S5 in Additional file 1 gives the probabilities of treatment, growth stage or their interaction having a significant effect on the abundance of each compound, and minimally adequate models chosen for determining significant differences in abundance among samples. The additional Tables S6, S7 and S8 in Additional file 1 give the fitted means, standard error of the difference and whether compounds differed significantly in abundance among variables of the minimally adequate models for the husk and caryopsis samples.

### Differences in compound abundance between growth stages

A number of compounds differed significantly in abundance between GS 75 and 77, independent of treatment (Additional file 1: Table S6). In the caryopsis samples, the abundance of tetracosanol, pentacosane, heptacosane and nonacosane was lower in GS 77 than 75. The fatty acids 9, 12-octadecadienoic acid and eicosanoic acid were present in increased amounts in GS 77 caryopses, as were the alkylresorcinols 5-heneicosylresorcinol, 5-pentacosyresorcinol and methyl-5-pentacosylresorcinol. The abundance of compound “Unknown-3” also increased between GS 75 and GS 77. In husk samples, the abundance of hexadecanol and hentriacontane was higher in GS 77 than GS 75. The abundance of tetradecanoic acid and branched 10-methylhexadecanoic acid also increased by GS 77. Esters and alkylresorcinols also increased in abundance in husk samples between GS75 and 77, specifically these were the following esters: docosyl tetradecanoate, eicosyl octadecanoate, docosyl tetracosanoate and the alkyresorcinols: 5-pentacosylresorcinol, methyl-5-pentacosylresorcinol and methyl-5-heptacosylresorcinol.

### Differences in compound abundance among treatments

The abundance of only a small number of compounds from caryopsis surface lipids changed significantly among treatments, independently of growth stage (Additional file 1: Table S7). Nonacosanol was present in greater abundance in caryopses from the WC treatment than those from the CC treatment. The abundance of hexacosyl octadecanoate was higher in CW caryopses compared with CC caryopses. Methyl-5-pentadecylresorcinol was more abundant in CC caryopses than in any other treatment, whereas methyl-5-heptadecylresorcinol was in greater abundance in CC caryopses than CW or WW. Nonacosan-14-one was more abundant in CC caryopses than WW.

A greater number of compounds from husk surface lipids had significant changes in abundance among treatments independent of growth stages compared with the caryopses. Docosanol was less abundant in CW husks than WW husks, whereas hexacosanol was less abundant in CW husks than WC husks. The fatty acids pentacosanoic acid and octacosanoic acid were more abundant in CC husks than CW husks, docosanoic acid was more abundant in the WW husks than either CC or CW husks, and the longer-chain hentriacontanoic acid was more abundant in WC husks that WW or CW. The abundances of nonacosan-14-one, 5-heptadecylresorcinol and methyl-5-heptadecylresorcinol were greatest in CC husks over all other treatments, with 5-pentadecylresorcinol being greatest in CC husks only compared with WC and CW husks. A large number of esters differed significantly in abundance among treatments, for all compound difference see Table S3 in Additional file 1. All identified esters derived from hexacosanol, known to be the most abundant barley surface lipid, had differences in abundance among treatments, with CC husks always having lower abundance of these esters than WW and WC husks, and WC having the highest abundance of these esters among all treatments although differences were not always significant. The compound “Unknown-4” had higher abundance in CC husks than all other treatments. “Unknown-6” had higher abundance in WW and WC husks than CC or CW husks.

### Differences in compound abundance where treatment and growth stage are significant factors

Those compounds where treatment and growth stage both have a significant effect on compound abundance, either individually or where the interaction is significant, are given in Additional file 1: Table S8. Within each of the four treatments, the abundance of hexadecanol increased significantly between GS 75 and 77 caryopses, but there were no differences in the abundance of hexadecanol among treatments within each growth stage. The WW caryopses had significantly more tricosanol than CC caryopses at GS 75, but at GS 77 WW caryopses had more tricosanol than both CC and CW caryopses. The abundance of both hexacosanol and octacosanol significantly decreased between GS 75 and GS 77 for all treatments, but there were no differences in abundance among the treatments within each growth stage. The same pattern was observed for octacosane, which decreased in abundance between GS 75 and 77, but was not different among treatments within each growth stage. At both GS 75 and 77, WW caryopses had a greater abundance of triacontane than CC caryopses. Both Unknown compounds 1 and 4 significantly increased in abundance from GS 75 to GS 77.

In the husk samples, the abundance of hexadecanoic acid was greater at GS 77 than GS 75 for all treatments. Similarly, the abundance of cholesterol was greatest at GS 77 than GS 75 for all four treatments. At GS 75, octacosyl hexadecanoate was more abundant in the CC and CW treatments than in WW husks, but at GS 77 the trend was reversed, with WW husks having significantly more of this ester than CC and CW husks. Hexacosanoic acid was more abundant in CW husks at both GS 75 and 77, than CC husks. Octadecananoic acid was less abundant in CC husks at GS 75 and 77 compared with WC husks.

### Differences in compound abundance where treatment and growth stage interact

The abundances of octadecanol, tritriacontane, campesterol and ß-sitosterol were significantly greater in the CW caryopses at GS 77 than in all other samples. Similarly, heptacosanol was most abundant in WW caryopses at GS 77 than all other samples. Pentacosanol was more abundant in WW caryopses at GS75 than in all other samples except for WC caryopses at GS 75. The alkenes hetacosene, nonacosene and hentriacontene were typically most abundant in CC caryopses at GS 75, than in all other samples. In the husk samples, octadecanol was significantly more abundant in CW organs at GS 77, corresponding to the same trend in the caryopses.

### Changes in surface lipid composition with respect to skinning

**Fig. 6:**
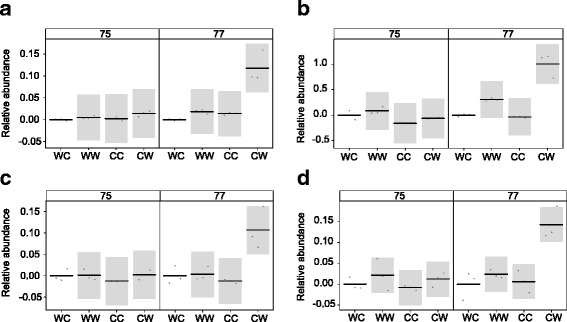
Contrast plots of model coefficients showing relative changes in abundance among treatments of the following compounds between GS 75 and 77 among the four treatments. **a** Octadecanol. **b** Tritriacontane. **c** Campesterol. **d** β-Sitosterol

As the WC plants had the highest proportion of skinned grains, and CW plants had the least proportion of skinned grains, compounds with significantly different levels of abundance between these treatments in the caryopsis extracts are of particular interest. Those compounds that change significantly at GS 77 in particular, when the caryopsis becomes sticky to the touch, might indicate which compounds are correlated with good quality husk adhesion. The CW caryopsis extracts at GS 77 have four compounds that increase significantly in abundance to all other samples. The estimated model coefficients (relative abundance to WC) and partial residuals for these compounds are shown in Fig. 6; octadecanol (Fig. 6a), tritriacontane (Fig. 6b), campesterol (Fig. 6c) and ß-sitosterol (Fig. 6d).

## Discussion

Grain skinning with different severities can be induced by imposing different temperatures during development of the husk and the caryopsis. Previous studies have reported that the critical production of sticky material on the caryopsis occurs from 10 days after anthesis [15, 17]. However, by relating developmental stages [44] and production of sticky material to the date of anthesis, we have shown that it is the developmental stage that is critical for husk adhesion, and that this can be reached over a wide range of days post-anthesis depending on the rate of development. Even the thermal time from anthesis until GS 77 varied widely among treatments, suggesting that pre-anthesis conditions also influence the development of the cementing layer by altering rate of grain development. As seen in previous studies using temperature to manipulate barley growth, similarly elevated temperatures shortened the period of the developmental stages to which they were applied and reduced the final grain weight [45, 58]. Ugarte et al. [14] found that short-term heat treatment pre-anthesis also decreased subsequent rates of grain development, although the present study shows that temperature during the grain filling period has a greater effect on the rate of grain filling than pre-anthesis temperatures, as the time from GS 65 to ripeness for the warm (CW and WW) or cool (CC and WC) post-anthesis conditions were more similar to each other, than treatments with the same pre-anthesis temperature. The temperature treatments that resulted in the most severe skinning levels (WC and WW), although not having a significant effect on the size of grain components, did have a marked effect on ear physiology. These treatments resulted in shorter ears with fewer fertilised florets developing to grains, and is likely due to heat stress at the time of anthesis in a similar manner to that reported in rice by Shi et al. [59]. A reduction in the number of grains produced per ear has previously been reported for barley and other grains grown at elevated temperatures [60]. It is likely that the low grain weights seen in the WW and CW treatments in the current study was due to a reduction in starch synthesis, as observed in other studies of elevated temperature during grain filling [45, 61].

Warm conditions during husk growth and carpel development resulted in higher skinning levels, with cool conditions during caryopsis development and grain filling further exacerbating skinning. It is unlikely that the different skinning levels were caused by an incompatibility between grain size and strength of the husk in this study as proposed by Rajasekaran et al. [7], as the weights and dimensions of husk components did not differ significantly among treatments once they had reached GS 77, and the treatment with the largest, heaviest grains at harvest (CC) did not correspond to the treatment with the most severe grain skinning. Only one variety was assessed in the current study however, examination of a number of diverse genotypes may find that skinning severity is correlated with varying husk and caryopsis dimensions in some varieties. Interestingly, the cool post-anthesis temperature of the WC treatment did not result in grains with a similar weight to those of CC plants, despite the similar grain filling duration of these two treatments. This result supports the finding of Calderini et al. [11] that higher temperature from booting to anthesis significantly reduces final grain weight. The effect of pre-anthesis temperature on grain weight has been assumed to be due to an effect on floret development [11, 14]; the results of the current study provide supporting data that this effect is independent of the size or weight of the husk, and therefore is probably due to development of the carpel, as reported in wheat by Xie et al. [62]. As the variation in skinning among the different treatments was not correlated with the size or weights of the grain components, it is likely that skinning was mediated through the effects that the different temperature treatments had on the cementing layer.

Until now, the most detailed study of the cementing layer has been completed by Gaines et al. [15]. Although others have since examined the cementing layer using light microscopy [16, 18] and electron microscopy [8]. The cementing layer is very thin, between 100 and 600 nm [15] and not reaching more than 120 nm in the present study. As the limit for resolution of light microscopy is reached at 200 nm, it is essential to use electron microscopy to gain the best understanding of differences in cementing layer structure. The structure of the pericarp and husk cuticles changed throughout grain filling in a manner consistent with the general description of cuticle development put forward by Jeffree [21, 63]. The pericarp cuticle at GS 75 had a reticulate boundary with the pericarp cell wall (Fig. 3a), consistent with continuing development of surface cuticles during cell wall expansion. Gaines et al. [15] described the cementing layer as a thin, electron-lucent layer with cell-wall material embedded in it, and suggested that it formed as early as 2 days after anthesis. However this description, and accompanying micrographs, correspond to what we currently know is a plant surface cuticle mid-development [21, 63], and may not necessarily correspond with production of cementing material as distinct from the pericarp cuticle. Indeed, a thin, electron-dense layer was visible on the outer surface of both the Nudinka and Concerto cuticles at GS 77 (Fig. 3d, Concerto not sown), similar to that described by Gaines et al. [15] as the cementing material. It was difficult to precisely distinguish boundaries between the pericarp surface cuticle and any cementing material until GS 85, when the husk and caryopsis adhere to each other and a layer with contrasting electron-lucency could be observed between the two cuticles as reported by Hoad et al. [64].

It is not clear from the results of this study, whether the lamellations seen in the cementing layer, when present, formed as part of the pericarp or husk cuticle, or whether they were only formed after contact of the glume with the caryopsis. These lamellations were often parallel, as observed by Gaines et al. [15], but in the regions of the greatest separation where the cementing material did not entirely fill the space between the husk and pericarp cuticle they became disordered. Typically, lamellations remained on the side of the pericarp at regions of separation, indicating that the cementing material is likely to be present on their surface, and that they are not part of the cementing layer itself. Extraction of eucalyptus spp. leaf cuticles with dichloromethane and ethanol resulted in lamellae becoming amorphous [20], indicating that such lamellae could be chemically distinct, although similar treatment of *Eucalyptus* spp. leaf cuticles left the lamellae intact [20]. Regions next to tubelike cells, most likely trichomes, always had some degree of separation between the husk and caryopsis. Gaines et al. [15] reported that tubelike cells did not produce a distinct cementing layer, and the cuticle of these cells in the present study was distinctly thinner than that of the surrounding pericarp epidermal cuticle. Although cuticle lamellations remained on the pericarp side, Gaines et al. [15] reported the cementing material always remained on the pericarp, the present study found that sometimes both the husk and the pericarp cuticle separated from the underlying cell wall. This demonstrates that the strength of adhesion between the two cuticles is greater than that between the surface cuticle and the cell wall, and suggests that the interface between the cuticle and the cell itself might be a point of weakness that influences skinning severity. Olkku et al. [16] reported that skinning was likely to be due to breakage of the large thin-walled parenchyma of the husk rather than separation along the cementing layer. Cell breakage was not observed in the present study, and may have been avoided by differences in sample preparation, however as husk thickness and the number of husk parenchyma cell layers were not measured in this study, we can not determine whether the different treatments had any effect on skinning in this way.

The composition of the surface lipids of the barley caryopsis has not yet been reported. However, Kakeda et al. [65] compared thin-layer chromatographs of surface lipids extracted from the caryopses of covered and naked barley, and found little evidence of solvent-extractable compounds being present on the naked barley caryopses, despite there being a thin cuticle present on naked barley caryopses. With the findings of Taketa et al. [18] that the cementing material could be dyed by Sudan Black dye, this indicates that the cementing material itself is likely to be extractable using organic solvents. The relative abundance of some compounds differed among treatments in relation to skinning severity in this study, in particular for the treatment with the lowest skinning (CW). The thickness of the cuticular layers and cementing layer is unlikely to have influenced the quality of husk-caryopsis adhesion among treatments in this study, suggesting that the ability of these cuticles to adhere to each other is significantly influenced by an altered composition rather than the thickness or amount of cuticular material.

Lack of correlation between cuticle thickness and physical properties is not unusual. Several studies have reported that plant cuticle composition is more correlated with physical or mechanical properties such as permeability and transpiration than cuticle size [66–69]. In this study, the triterpenoids and their derivative sterol compounds, particularly ß-sitosterol and campesterol had the most strikingly altered increase in abundance at GS 77 among treatments that resulted in different skinning severities. Some plants with higher proportions of triterpenoids in their cuticles have higher cuticle permeability [66], and mutants with altered surface lipid compositions that result in higher cuticle permeability often display an organ fusion phenotype, particularly among floral organs. The long-chain alkane tritriacontane was also associated with good quality husk adhesion; *n*-alkanes have also been linked to cuticular permeability [66], however in tomato fruits, higher-chain alkanes with C_31_ to C_34_ were associated with lower cuticle permeability [67]. Organ fusion has been previously noted to be a similar phenotype to husk adhesion [43]. In the maize *ad1* mutant, where the cell walls of floral parts adhere to each other, the cuticle appears to remain intact between the cell walls [41], similar in appearance to the cementing layer comprising the husk and pericarp surface cuticles. In the case of the *Arabidopsis* mutants *atwbc11* and *bdg* (*bodyguard*) however, the cuticle is disrupted and the cell walls merge [40, 70]. Neither disruption of cuticular layers or direct adhesion of husk and pericarp cell walls was observed in the present study. Organ fusion has been induced by expression of a fungal cutinase in *Arabidopsis*, where cuticular layers either remaned intact between cell walls, or walls of epidermal cells came in direct contact with each other [71]; organ fusion has also been induced by repression of *WIN1/SHN1*, a gene with homology to *Nud*, in *Arabidopsis* and *Tourenia fournieri* [72]. The increase in abundance of compounds known to increase cuticle permeability in samples with the highest quality husk adhesion, supports the observations of Duan et al. [43] that the process of husk adhesion is similar to organ fusion, and may be due to an increase in cuticle permeability. Although the present study suggests that cuticular wax composition has a role in the quality of husk adhesion, it is possible that the entire matrix of cell-wall, cutin and cuticular waxes contribute to husk adhesion.

## Conclusions

In this study, we were able to determine that adhesion of the barley husk to the underlying caryopsis is directly dependent on the developmental stage of the grains regardless of the rate of grain development. It is not until the caryopsis nears maximum volume (GS 77) that the adhesive cementing layer comes into contact with the husk and adhesion is initiated. Therefore it is vital to account for developmental variation in future studies of husk adhesion, and not just the number of days post-anthesis. We now have evidence that the composition of the cuticular waxes on the caryopsis influences the quality of husk adhesion, and therefore has a tangible effect on the severity of the malting quality defect grain skinning. The quality of the husk adhesion is influenced by environmental conditions, but it is likely that there is a genetic influence on husk adhesion, as we know that different cultivars of malting barley have differential susceptibility to grain skinning [4]. As the current work has indicated that differences in skinning risk are likely due to cuticle composition, rather than differences in grain dimensions among cultivars, research efforts to minimise skinning risk in future cultivars should focus on determining the genetic controls of cementing layer composition.

## Additional files

Additional file 1: Is an excel workbook containing 8 sheets, with supplementary tables of data or analysis results. These are numbered **Table S1** through **Table S8**, and are each invididually referred to in the tex as, for example “...shown in **Table S2**”. (XLSX 301 kb)

Additional file 2: Is a schematic of mass spectrometric fragmentation patterns to enable readers to understand how certain lipid compounds were identified using their characteristic fragmentations. (PDF 75 kb)

Additional file 3: **Figure S4** a is a light micrograph of a resin-embedded barley grain at GS 85 stained with 1% (*w*/*v*) Toluidine bue O and counter-dyed with 1:1 0.1% (*w*/*v*) Sudan Red in polyethylene glycol:90% (*v*/v) glycerol. The interface between the pericarp epidermis and the husk (where the cementing layer is present) is shown (black arrow). The testa cuticle has dyed with Sudan Red (black and white arrow). The aleurone layer (black and gray arrow) marks the beginning of the endosperm. **b** shows the separation of the pericarp cuticle (black arrow), exposing the underlying pericarp cell wall (black and white arrow). **c** is a high-resolution of the smooth surface of the Nudinka pericarp at GS 77. There is no evidence of a cementing material, and no damange to the cuticle surface. (PDF 483 kb)

Additional file 4: Is a large series of graphs showing the mean abundance of compounds for each structural class, ordered by carbon-number, for each treatment and growth stage sampled. Some readers may be interested in compound distributions as displayed this way, although we do not feel it was essential to the main message of the text. (PDF 97 kb)

Additional file 5: **Figure S3** Interrelationship between Hentriacontan-14,16-dione and enol and (enol)2 tautomers of Hentriacontan-14, 16-dione. (PDF 30 kb)

## Abbreviations

ANOVA: Analysis of variance
CC: Cool, cool treatment
CW: Cool, warm treatment
GS: Growth stage
TMS: Trimethylsilyl
WC: Warm, cool treatment
WW: Warm, warm treatment
