# Supplementary material for: Husk to caryopsis adhesion in barley is influenced by pre- and post-anthesis temperatures through changes in a cuticular cementing layer on the caryopsis
Source: BMC Plant Biol. 2017 Oct 23;17:169. doi: 10.1186/s12870-017-1113-4 (PMC5651604; doi:10.1186/s12870-017-1113-4)
Supplement: Supplementary file 2 — Is a schematic of mass spectrometric fragmentation patterns to enable readers to understand how certain lipid compounds were identified using their characteristic fragmentations. (PDF 75 kb) [file 12870_2017_1113_MOESM2_ESM.pdf]

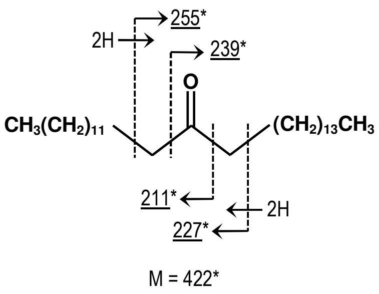

Nonacosan-14-one

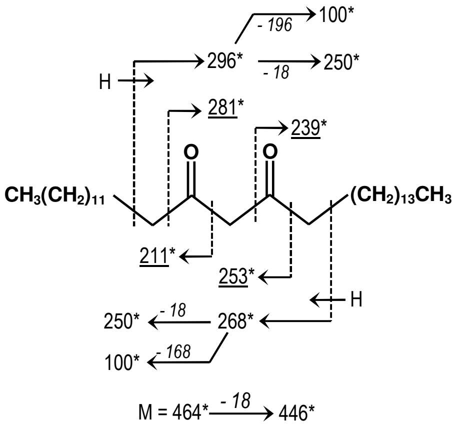

Hentriacontan-14,16-dione

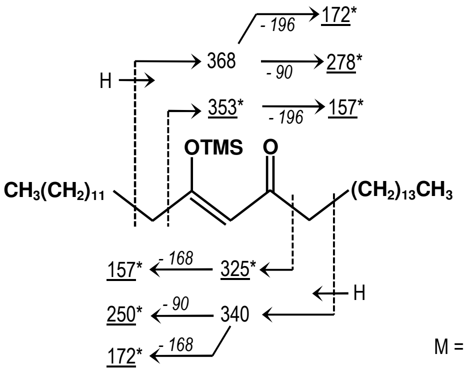

M = 536* - 15 → 521*

Hentriacontan-14-en-14-ol-16-one (TMS)

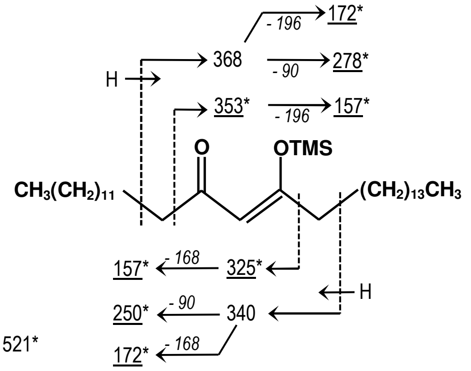

Hentriacontan-14-one-15-en-16-ol (TMS)

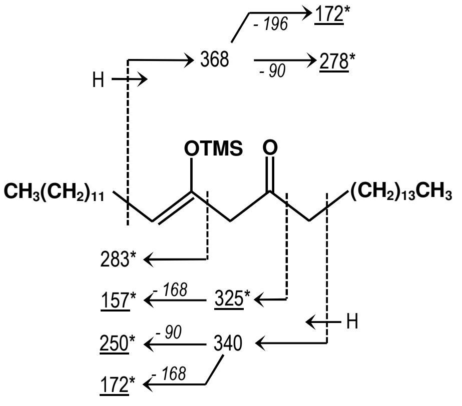

Hentriacontan-13-en-14-ol-16-one (TMS)

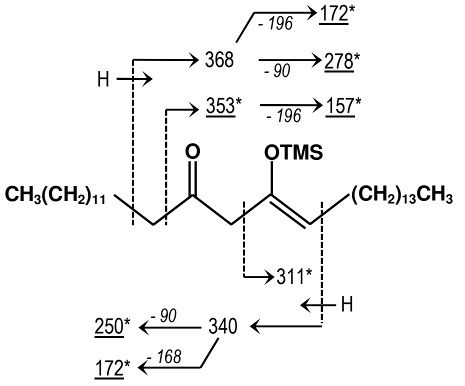

Hentriacontan-14-one-16-en-16-ol (TMS)

M = 608* -15 → 593*

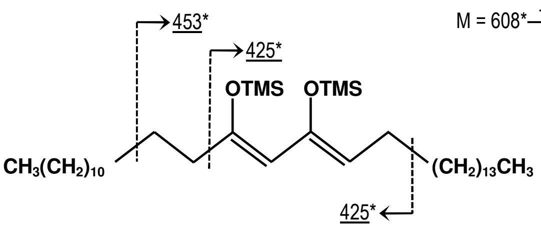

Hentriacontan-14, 16-dien-14, 16-diol $(TMS)_2$

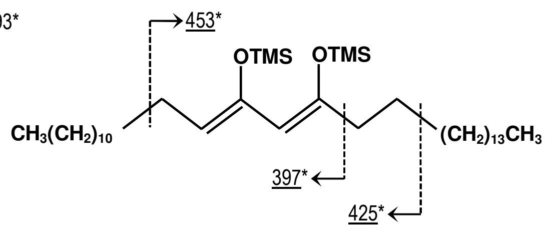

Hentriacontan-13, 15-dien-14, 16-diol $(TMS)_2$

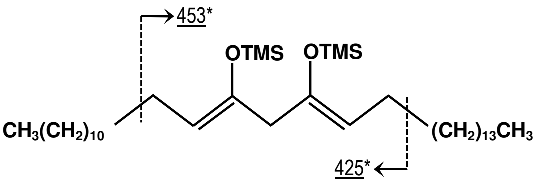

Hentriacontan-13, 16-dien-14, 16-diol $(TMS)_2$

M = 552* -15 → 537*

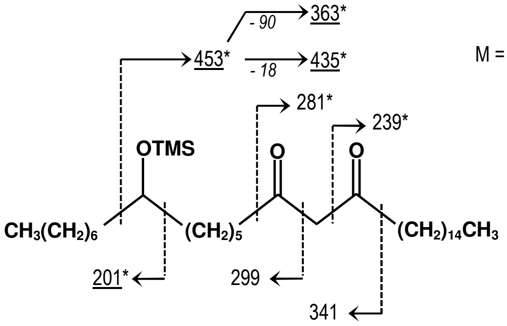

Hentriacontan-8-ol -14,16-dione (TMS)

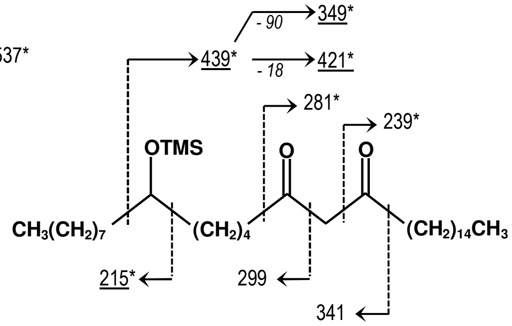

Hentriacontan-9-ol -14,16-dione (TMS)

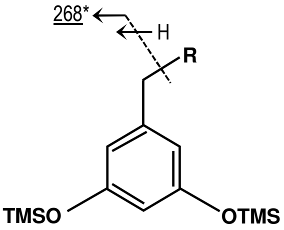

5-Alkylresorcinols $(TMS)_2$

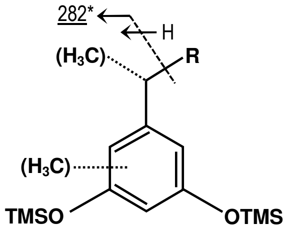

Methyl-5-Alkylresorcinols $(TMS)_2$

Figure S1. Mass spectrometric fragmentation patterns for ketones and TMS derivatives of β-diketones, enol and $(enol)_2$ tautomers of β-diketones, hydroxyl-β-diketones and 5-alkyl resorcinols found in cuticular waxes of barley caryopses and husk.
