# Supplementary material for: Husk to caryopsis adhesion in barley is influenced by pre- and post-anthesis temperatures through changes in a cuticular cementing layer on the caryopsis
Source: BMC Plant Biol. 2017 Oct 23;17:169. doi: 10.1186/s12870-017-1113-4 (PMC5651604; doi:10.1186/s12870-017-1113-4)
Supplement: Supplementary file 3 — Figure S4 a is a light micrograph of a resin-embedded barley grain at GS 85 stained with 1% (w/v) Toluidine bue O and counter-dyed with 1:1 0.1% (w/v) Sudan Red in polyethylene glycol:90% (v/v) glycerol. The interface between the pericarp epidermis and the husk (where the cementing layer is present) is shown (black arrow). The testa cuticle has dyed with Sudan Red (black and white arrow). The aleurone layer (black and gray arrow) marks the beginning of the endosperm. b shows the separation of the pericarp cuticle (black arrow), exposing the underlying pericarp cell wall (black and white arrow). c is a high-resolution of the smooth surface of the Nudinka pericarp at GS 77. There is no evidence of a cementing material, and no damange to the cuticle surface. (PDF 483 kb) [file 12870_2017_1113_MOESM3_ESM.pdf]

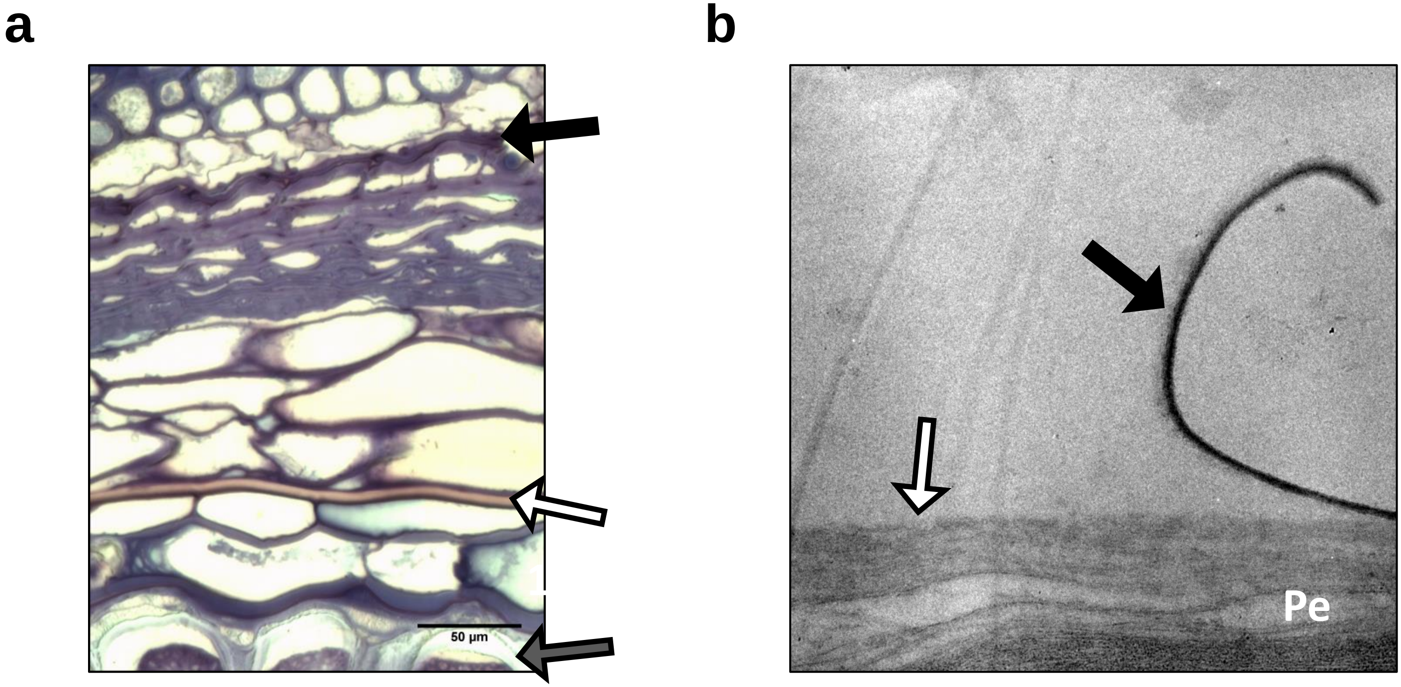

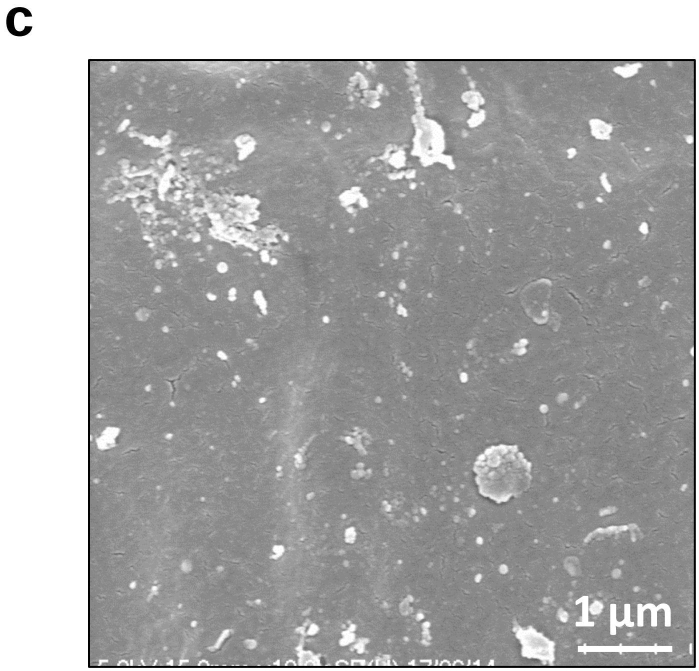

Figure S4. a is a light micrograph of a resin-embedded barley grain at GS 85 stained with 1% (w/v) Toluidine bue O and counter-dyed with 1:1 0.1% (w/v) Sudan Red in polyethylene glycol:90% (v/v) glycerol. The interface between the pericarp epidermis and the husk (where the cementing layer is present) is shown (black arrow). The testa cuticle has dyed with Sudan Red (black and white arrow). The aleurone layer (black and gray arrow) marks the beginning of the endosperm. **b** shows the separation of the pericarp cuticle (black arrow), exposing the underlying pericarp cell wall (black and white arrow). **c** is a high-resolution of the smooth surface of the Nudinka pericarp at GS 77. There is no evidence of a cementing material, and no damange to the cuticle surface.
