# Supplementary material for: Husk to caryopsis adhesion in barley is influenced by pre- and post-anthesis temperatures through changes in a cuticular cementing layer on the caryopsis
Source: BMC Plant Biol. 2017 Oct 23;17:169. doi: 10.1186/s12870-017-1113-4 (PMC5651604; doi:10.1186/s12870-017-1113-4)
Supplement: Supplementary file 5 — Figure S3 Interrelationship between Hentriacontan-14,16-dione and enol and (enol)2 tautomers of Hentriacontan-14, 16-dione. (PDF 30 kb) [file 12870_2017_1113_MOESM5_ESM.pdf]

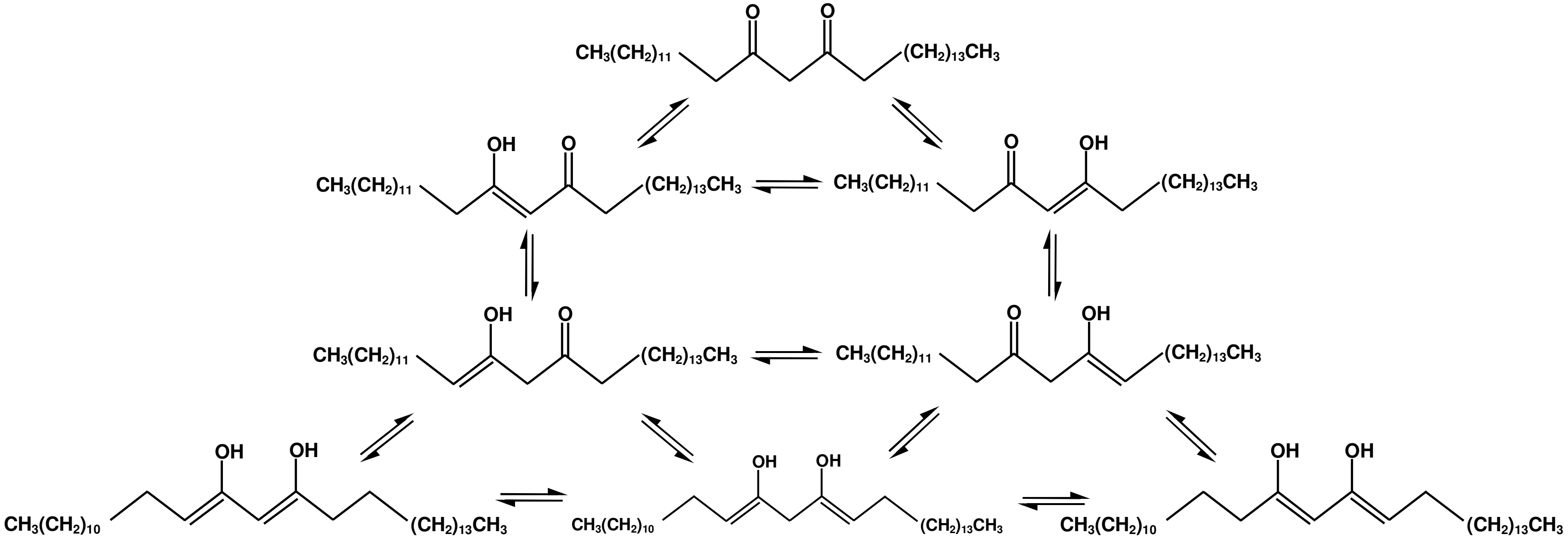

Figure S3. Interrelationship between Hentriacontan-14,16-dione and enol and $(enol)_2$ tautomers of Hentriacontan-14,16-dione
