# Supplementary material for: Husk to caryopsis adhesion in barley is influenced by pre- and post-anthesis temperatures through changes in a cuticular cementing layer on the caryopsis
Source: BMC Plant Biol. 2017 Oct 23;17:169. doi: 10.1186/s12870-017-1113-4 (PMC5651604; doi:10.1186/s12870-017-1113-4)
Supplement: Supplementary file 4 — Is a large series of graphs showing the mean abundance of compounds for each structural class, ordered by carbon-number, for each treatment and growth stage sampled. Some readers may be interested in compound distributions as displayed this way, although we do not feel it was essential to the main message of the text. (PDF 97 kb) [file 12870_2017_1113_MOESM4_ESM.pdf]

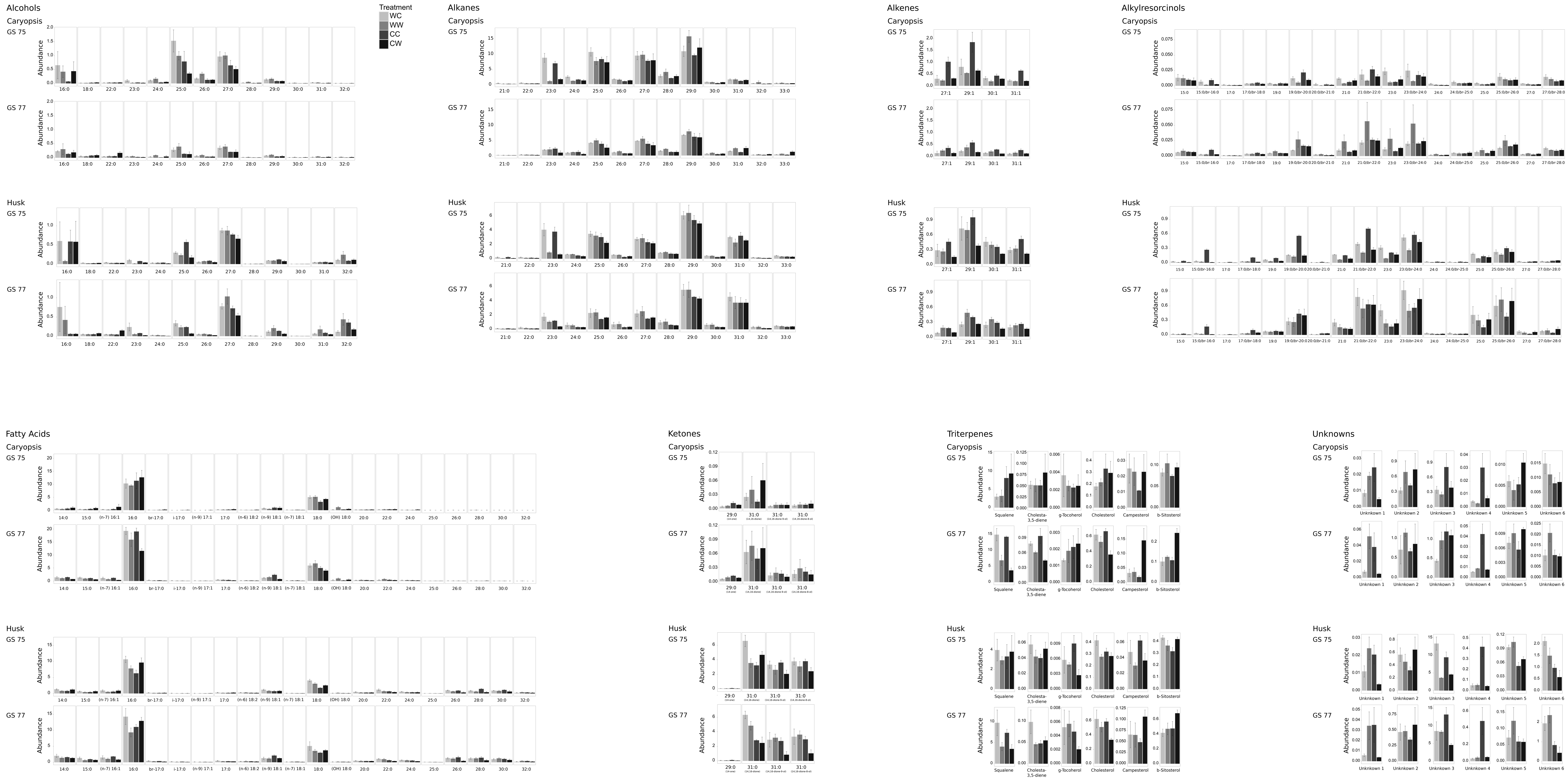

Figure S2. Abundance of individual compounds by carbon number for each compound class (esters not shown). Error bars are +/- standard error of the mean. Refer to Table S1 for compound names, and the ions selected for quantification.
